# Combined heart and liver transplantation for homozygous familial hypercholesterolaemia: 35-year post-operative outcomes

**DOI:** 10.21542/gcsp.2026.38

**Published:** 2026-08-31

**Authors:** Rona Jalandari, Fatima Omar, Fawzi Lazem, Mahmoud Barbir

**Affiliations:** 1 Harefield Hospital, Guy’s and StThomas’ NHS Foundation Trust, London, United Kingdom; 2 Magdi Yacoub Institute, Harefield, Middlesex, United Kingdom

## Abstract

Combined heart–liver transplantation (CHLT) is an exceptionally rare multi-organ procedure undertaken in patients with concurrent end-stage cardiac and hepatic disease, or in those with inherited metabolic disorders—most notably homozygous familial hypercholesterolaemia (HoFH)—in which defective hepatic LDL receptor (LDL-R) function drives progressive and otherwise irreversible cardiovascular injury. We report a 35-year follow-up of a female patient who underwent simultaneous orthotopic cardiac and liver transplantation on 13 September 1990 for HoFH complicated by severe ischaemic cardiomyopathy and aortic valve stenosis. Pre-operative serum low-density lipoprotein cholesterol (LDL-C) was 13 mmol/L, refractory to all pharmacological interventions available at the time. Transplantation produced immediate normalisation of LDL-C to 2.1 mmol/L, reflecting instantaneous restoration of donor hepatic LDL-R function—consistent with the landmark observations reported by Bilheimer, Goldstein, Brown, and Starzl^1, 2^. This normalisation has been broadly maintained across three and a half decades of follow-up. Lipid-lowering therapy was commenced post-operatively to augment the normalised lipid profile, and dramatic regression of tendon xanthomata was documented within six months of transplantation^11^. Cardiac graft function has remained preserved, with ejection fraction (EF) of 64–68% on recent echocardiography and no inducible ischaemia on sequential myocardial perfusion scintigraphy (MPS) in May 2025. Progressive cardiac allograft vasculopathy (CAV) has been identified on coronary CT angiography (CTCA) in 2024 but without haemodynamic consequence. Rejection burden across 35 years has been remarkably low—comprising a mild episode at three weeks post-transplant and a single biopsy-confirmed Grade 1R episode in 2013—consistent with the hepatic immunological privilege hypothesis first elaborated by Calne and subsequently substantiated in combined organ transplantation literature^3, 4^. The dominant long-term complication has been end-stage renal failure secondary to calcineurin inhibitor (CNI) nephropathy—a well-recognised sequela of long-term CNI exposure^27^—necessitating haemodialysis via arteriovenous fistula. More recently, the patient sustained a low-impact femoral fracture reflecting steroid-associated osteoporosis, with subsequent prolonged hospitalisation, deconditioning, rotator cuff tear, and bilateral upper limb neuromuscular symptoms under active investigation. This case contributes exceptional 35-year outcome data in a field where long-term follow-up beyond ten to fifteen years remains sparse.

## Introduction

Familial hypercholesterolaemia (FH) is the clinical manifestation of genetic lesions affecting the LDL receptor pathway, most commonly mutations in the gene encoding the LDL receptor itself^5, 6^. The condition is characterised by markedly elevated circulating LDL-C and premature, aggressive atherosclerotic cardiovascular disease, including coronary artery disease and left ventricular outflow tract obstruction^7^. The severity of the clinical syndrome is determined both by the underlying genetic lesion—homozygotes being affected earlier and more profoundly than heterozygotes—and by the adequacy of therapeutic intervention^5^.

Although multiple pharmacological strategies exist to reduce LDL-C in heterozygous FH, none achieves sufficient reduction in patients with biallelic LDL-R loss-of-function mutations where receptor activity is near-absent. The agents available in the early 1990s—bile acid sequestrants, niacin, probucol, and lipoprotein apheresis—were wholly dependent on residual receptor activity or provided only transient benefit, as elegantly demonstrated by Hoeg, Starzl, and Brewer in their landmark 1987 comparison of hepatic transplantation, lipid-lowering medication, and plasma exchange in HoFH^8^. Liver transplantation provides the definitive source of functional LDL-Rs, with up to 80% of the body’s LDL-Rs residing in the hepatic parenchyma; liver transplantation alone is therefore curative for the metabolic defect^1, 9^. In patients who have additionally developed end-stage ischaemic cardiomyopathy from decades of uncontrolled hypercholesterolaemia, CHLT addresses both the metabolic substrate and the cardiac consequence simultaneously.

Beyond metabolic correction, CHLT confers an important immunological advantage through the phenomenon of hepatic immunological privilege. The liver allograft absorbs donor-specific antibodies (DSA), downregulates alloreactive T-cell responses, and creates a tolerogenic immune milieu that attenuates both humoral and cellular rejection of the co-transplanted cardiac graft^3, 10^. Sir Roy Calne first documented this in pigs; Kamada and colleagues subsequently confirmed it in rat models^3, 4^. In clinical practice this is manifest as lower rates of cardiac rejection and reduced immunosuppressive requirements in CHLT recipients compared with isolated cardiac transplant recipients.

CHLT remains exceptionally rare globally. United Network for Organ Sharing (UNOS) registry data identify 532 adult procedures between 1990 and 2023 across 46 United States centres, with contemporary 1-, 3-, and 5-year survival rates of 85%, 80%, and 77% respectively. In the United Kingdom, only five CHLTs were performed in the decade between April 2011 and March 2021, during a period when 1,443 isolated heart transplants were undertaken. The present case, performed on 13 September 1990, represents one of the earliest documented adult CHLTs in the UK and—at 35 years post-operatively—one of the longest-surviving such patients in the world. Its translational lessons, from molecular lipid biology to operational immunological tolerance and long-term multi-system comorbidity, extend and build upon those first articulated by Ibrahim, El-Hamamsy, Barbir, and Yacoub at the 20-year mark^11^.

## Case description

### Patient demographics and initial presentation

**Table:** 

**Patient demographics and operative characteristics**	
Age at transplant	33 years (born 1957; currently 69 years of age)
Sex	Female
Date of operation	13 September 1990
Procedure	Simultaneous orthotopic cardiac and hepatic transplantation
Primary indication	Homozygous familial hypercholesterolaemia (HoFH) with severe ischaemic cardiomyopathy and aortic valve stenosis
Pre-operative LDL-C	13 mmol/L —refractory to all available lipid-lowering pharmacotherapy
Pre-operative total cholesterol	23 mmol/L (pre-treatment); 14.4 mmol/L (on simvastatin + cholestyramine)
Genetic findings	Compound heterozygosity at LDL-R locus: large deletion on one allele; missense mutation on second allele producing a receptor with delayed membrane transport and ∼10% of normal LDL-R activity^11^
Clinical stigmata	Diffuse cutaneous and tendon xanthomata; bilateral carotid bruits; severe diffuse coronary artery disease; aortic stenosis; LVOT obstruction^11^

### Family history

**Figure 1.**
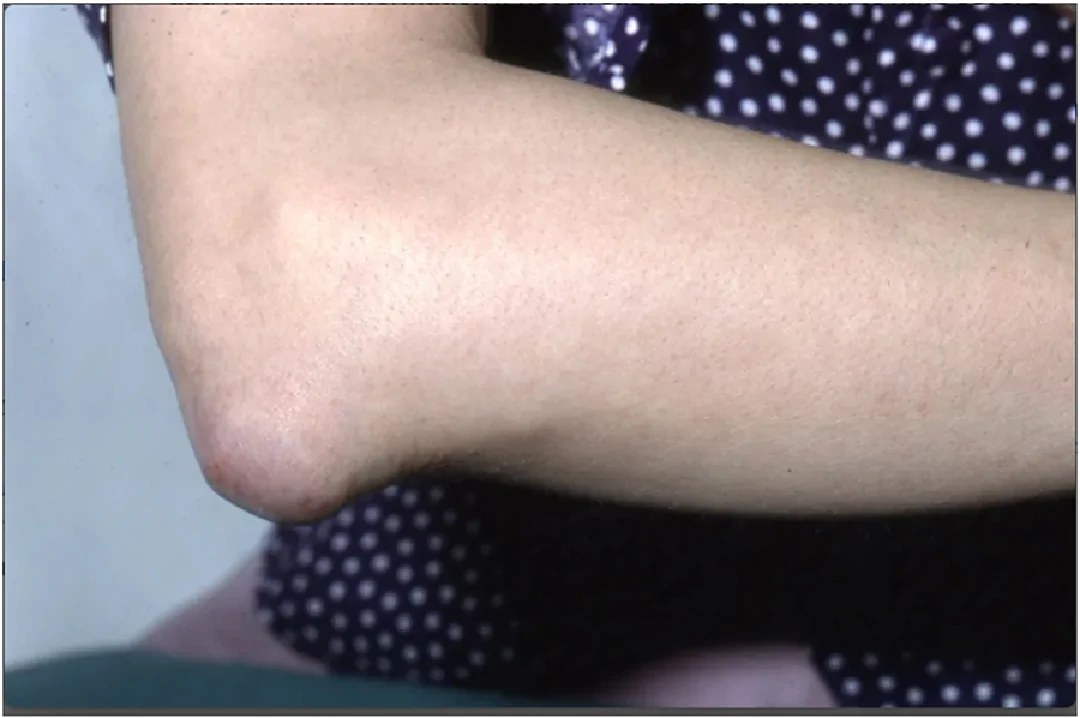
Tendon xanthomata at presentation, demonstrating extravascular lipid deposition characteristic of severe homozygous familial hypercholesterolaemia.

The patient was known to have severe hypercholesterolaemia from childhood. There is a strong family history of inherited dyslipidaemia: her brother, aged 31 years, is also known to have hypercholesterolaemia, and both parents are known to have hypercholesterolaemia, having sustained myocardial infarctions at the ages of 51 and 59 years respectively. This pedigree is consistent with biallelic inheritance of LDL-receptor dysfunction underlying the patient’s homozygous familial hypercholesterolaemia (Figure 1).

### Indication for combined heart–liver transplantation

This patient presented in severe heart failure in 1990 with advanced atherosclerotic disease resulting in significant coronary artery disease and aortic valve stenosis. As described in the original case report by Ibrahim et al.^11^, the serum LDL-C of 13 mmol/L was explicitly refractory to conventional lipid-lowering therapy. Genetic analysis confirmed compound heterozygosity at the LDL receptor locus, with a large deletion on one allele and a missense mutation producing a receptor with approximately 10% of normal LDL-R activity. The combined genotype resulted in near-absent physiological LDL clearance.

Therapeutic strategies trialled prior to transplantation—bile acid sequestrants (cholestyramine, colestipol), nicotinic acid, probucol, and lipoprotein apheresis—are reviewed in the context of their mechanisms and limitations in HoFH. Bile acid sequestrants interrupt enterohepatic bile acid recirculation to upregulate LDL-R expression; in HoFH, where receptor activity is near-absent, responses rarely exceed 10–15%^11^. Nicotinic acid achieves LDL-C reductions of 20–30% in heterozygous FH via reduced hepatic VLDL synthesis, but is limited by poor tolerability and inadequate effect in near-homozygous receptor deficiency. Probucol reduces LDL-C by approximately 10–15% via an LDL-R-independent mechanism and was valued for promoting xanthoma regression, but produced clinically insufficient reduction from a baseline of 13 mmol/L. Lipoprotein apheresis achieves acute 50–70% LDL-C reductions per session but requires weekly procedures indefinitely, as the dysfunctional native liver fails to prevent rapid reaccumulation. Regarding statin therapy, lovastatin had only recently entered clinical practice in 1990. It upregulates LDL-R expression through HMG-CoA reductase inhibition but requires functional receptors; this patient’s biallelic dysfunction rendered statin monotherapy insufficient.

Hoeg, Starzl, and Brewer demonstrated in 1987 that liver transplantation was substantially superior to both medication and plasma exchange for achieving sustained LDL-C normalisation in HoFH^8^, providing the scientific rationale for the CHLT strategy employed in this case. Given the patient’s compound LDL-R deficiency and severe cardiac failure, combined heart–liver transplantation was undertaken both as life-saving cardiac replacement and as definitive metabolic therapy, replacing the dysfunctional hepatic LDL-R with a fully functional donor allograft.

### Peri-operative course

**Figure 2.**
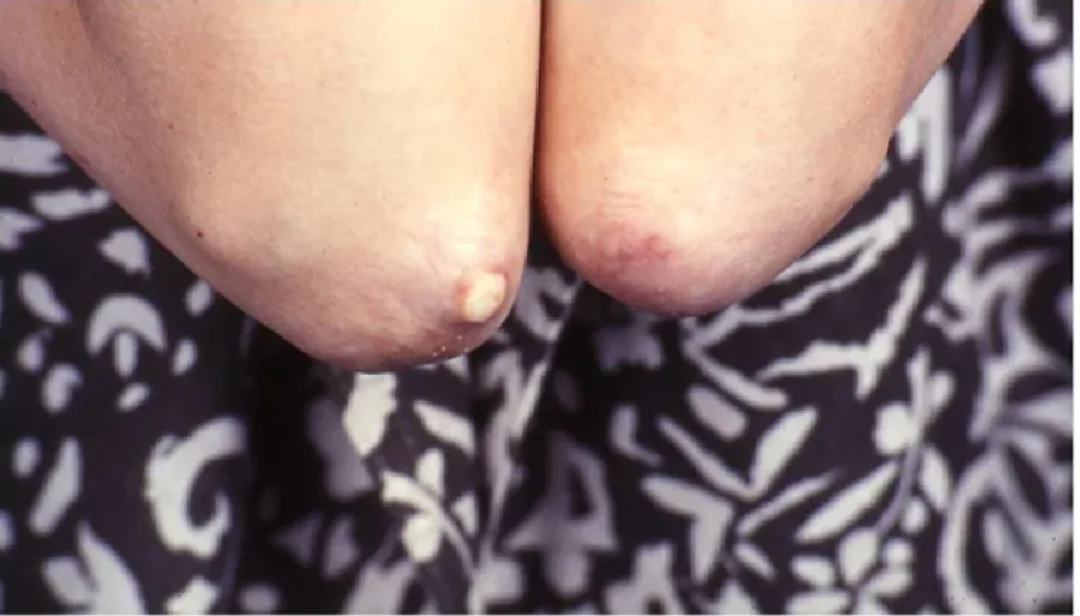
Appearance at six months post-transplantation, demonstrating marked regression of tendon xanthomata following sustained normalisation of LDL-C.

The patient underwent simultaneous orthotopic cardiac and hepatic transplantation on 13 September 1990. As documented in the original case report^11^, immediate post-operative lipid assessment demonstrated rapid normalisation of serum LDL-C to 2.1 mmol/L at ten days post-operatively, reflecting the immediate functional contribution of the donor hepatic LDL-R. Total cholesterol correspondingly fell from 14.4 mmol/L (on pre-operative lipid-lowering therapy) to 3.4 mmol/L. Early post-operative recovery was complicated by a mild episode of cardiac rejection at three weeks post-transplant, managed with intensive prednisolone therapy and resolved fully. Dramatic regression of tendon xanthomata was documented by six months following transplantation, consistent with the known correlation between sustained LDL-C reduction and extravascular lipid clearance^11^ (Figure 2).

## Long-Term Follow-Up: 35-Year Summary

### 1. Metabolic response: lipid profile

Transplantation produced immediate and durable correction of the patient’s dyslipidaemia. Pre-operatively, the serum LDL-C was 13 mmol/L (total cholesterol 14.4 mmol/L on lipid-lowering therapy), reflecting near-absent LDL receptor function. At ten days post-transplant, LDL-C had normalised to 2.1 mmol/L and total cholesterol to 3.4 mmol/L, consistent with the values reported by Bilheimer et al. following hepatic replacement^1, 2^. Serial lipid measurements across twenty years were reported by Ibrahim et al. in 2012, demonstrating sustained LDL-C stability^11^. The historical lipid trajectory is summarised below.

**Table:** 

**Lipid parameter**	**Pre-CHLT (on therapy)**	**10 days post-CHLT**	**20 years post-CHLT [12]**
Total Cholesterol (mmol/L)	14.4	3.4	6.3
LDL-C (mmol/L)	13.0	2.1	4.3
HDL-C (mmol/L)	0.88	0.44	1.0
Triglycerides (mmol/L)	1.2	1.9	2.1
Lipid-lowering therapy	Simvastatin + cholestyramine	None	Atorvastatin

**Current lipid management (2026):** Atorvastatin 10 mg once daily; Ezetimibe 10 mg once daily (under lipid specialist review). Persistent cholesterol elevation noted on bloods at May 2025 clinic review. Late residual hypercholesterolaemia likely reflects secondary contributions from ciclosporin-induced dyslipidaemia and chronic renal failure, rather than recurrence of the primary genetic defect (see Discussion).

### 2. Cardiac graft function and surveillance

Cardiac graft function has been preserved across the follow-up period. Trans-thoracic echocardiography at twenty years demonstrated an ejection fraction of 68% with fractional shortening of 30%, and a coronary calcification score of 0^11^. Subsequent surveillance has documented maintained systolic function, with EF of 68% confirmed in July 2024 and 64% at the focused echocardiographic assessment at the clinic review of 14 May 2026. Myocardial perfusion scintigraphy in May 2025 demonstrated a normal-sized left ventricle with preserved EF of 75% and no evidence of stress-induced perfusion abnormality.

Progressive cardiac allograft vasculopathy has been identified on CTCA in July 2024, demonstrating increasing calcification in the LAD, increasing stenosis in the ostial circumflex artery, and mild stenosis in the RCA and left main stem. Importantly, sequential MPS in October 2022 and May 2025 has confirmed the absence of haemodynamically significant inducible ischaemia, and preserved systolic function indicates adequate functional cardiac reserve. HLA antibody testing in May 2025 confirmed no donor-specific antibodies. A new symptom of atypical chest discomfort—burning and tightness occurring in the early morning and when recumbent, relieved by sitting forwards, without exertional correlation—was reported at the May 2026 clinic review and was not considered consistent with angina at this time.

**Table:** 

**Investigation/Date**	**Findings**
Echo 19/03/2013	LVEF 67%; normal baseline function
Echo 12/09/2013	LVEF 53% (decline associated with acute rejection episode)
Echo 18/09/2013	LVEF 50% (nadir during rejection)
Echo 07/2024	LVEF 68%; normal RV size and function; normal filling pressures —full recovery to baseline
Echo 14/05/2026 (focused)	EF 64% —preserved systolic function confirmed at most recent clinic review
MPS 10/2022	No inducible ischaemia
MPS 05/2025	Normal-sized left ventricle; preserved EF 75%; no evidence of stress-induced perfusion abnormality
CTCA 07/2024	Progressive calcification in LAD; increasing stenosis in ostial circumflex artery; mild stenosis in RCA and left main stem —consistent with progressive CAV
HLA 11/2021	HLA antibody negative
HLA 07/2024	HLA positive; no donor-specific antibodies (DSA) identified
DSA 12/05/2025	DSA negative
Chest symptoms (05/2026)	Burning and tightness; early morning/positional onset; 10 min; relieved by sitting forwards; atypical of angina. Repeat MPS deferred; telephone review in 1 month.

### 3. Rejection history

The patient’s rejection burden across 35 years has been remarkably low, comprising two documented episodes and one presumed episode, consistent with the hepatic immunological privilege of CHLT. The original case report noted the absence of rejection despite cyclosporin trough levels substantially below those considered standard for isolated cardiac transplantation^11^.

• **Week 3 post-transplant:** Mild episode of cardiac rejection at 3 weeks post-transplant (1990). Managed with intensive prednisolone therapy; full recovery^11^.
• **September 2013:** Biopsy-confirmed Grade 1R rejection on sirolimus-based immunosuppression (September 2013, 23 years post-transplant). Treated with pulse methylprednisolone; conversion to ciclosporin. EF subsequently recovered from the nadir of 50% back toward the pre-rejection baseline of 67% (recorded 19 March 2013).
• **June 2014:** Breathlessness, orthopnoea, reduced EF, and reduced ECG voltage. Biopsy negative for rejection; however, clinical improvement after three days of pulse methylprednisolone was interpreted as consistent with a false-negative biopsy result (June 2014).

### 4. Hepatic graft function

Liver graft function has been consistently excellent throughout the follow-up period. Liver function tests have remained normal since transplantation^11^, with no documented hepatic decompensation, biliary complications, or graft failure. Hepatology clinic records confirm no evidence of significant fibrosis. LFTs are monitored in parallel with ciclosporin trough levels as part of ongoing immunosuppression management.

### 5. Complications over follow-up

#### End-stage renal failure (CNI nephropathy)

The most significant long-term complication has been progressive renal impairment secondary to calcineurin inhibitor nephropathy from long-term cyclosporin exposure. The original case report documented a progressive rise in serum creatinine from 135 *μ*mol/L (post-operative baseline) to 285 μmol/L by late 2009, despite maintained cyclosporin doses substantially lower than those used in isolated cardiac transplantation^11^. Ibrahim et al. noted that substitution of cyclosporin for sirolimus and MMF in 2007 might reduce the rate of decline, citing evidence from Lyster et al.^11, 13^. Ultimately, end-stage renal failure was established, and the patient commenced permanent haemodialysis via a right brachiocephalic arteriovenous fistula (Tuesday, Thursday, and Saturday schedule). The fistula has been complicated by aneurysmal enlargement and a haemorrhage requiring surgical revision. A current fistula site infection on the lower right arm is being treated with flucloxacillin.

#### Cardiac allograft vasculopathy

Progressive CAV has been documented on CTCA in 2024, with increasing LAD calcification, ostial circumflex stenosis, and mild RCA and left main stem stenosis. Sequential MPS in 2022 and 2025 has not demonstrated haemodynamically significant ischaemia, and preserved EF on echocardiography is reassuring. CAV surveillance with non-invasive imaging is continued. The atypical chest symptoms reported in May 2026—positional, non-exertional, short-lived, relieved by sitting forwards—were considered more consistent with a musculoskeletal or gastrointestinal aetiology than angina at clinic review, with telephone review planned in one month.

#### Skeletal morbidity: osteoporosis and femoral fracture

Chronic exposure to corticosteroids and calcineurin inhibitors accelerates bone loss through reduced osteoblast activity, increased osteoclast-mediated resorption, impaired calcium handling, and direct toxicity to bone microarchitecture^14, 15^. In late 2024, this patient sustained a low-impact fracture of the right femur following a fall. Conservative management was undertaken due to concerns regarding wound infection risk in the immunosuppressed state. A prolonged admission of approximately three months was complicated by hospital-acquired pneumonia and significant deconditioning. Following discharge home with a care package, the patient now mobilises with a walker and requires carers three times per week. Left hip pain attributed to osteoporosis prevents independent ambulation, and an MDT decision has determined that hip replacement would carry excessive risk. Referral to the Pain Team and Metabolic Bone Team has been arranged, and steroid joint injections have been confirmed as safe in the context of current immunosuppression.

#### Upper limb neuromuscular symptoms

Following discharge from the prolonged hospital admission, the patient developed progressive bilateral arm and hand weakness and numbness, significantly impairing feeding and self-care. There is no associated back pain or lower limb neurological symptoms. A rotator cuff tear and frozen shoulder of the right shoulder have also been diagnosed, for which physiotherapy has been commenced. The aetiology of the bilateral upper limb neuropathy remains under active investigation. The differential includes compressive neuropathy (exacerbated by walker use), uraemic neuropathy in the context of dialysis-dependent renal failure, cervical myelopathy, and dialysis-related amyloid arthropathy.

#### Carotid atherosclerosis and prior cerebrovascular accident

The patient has a documented history of prior cerebrovascular accident. Carotid Doppler ultrasonography performed in June 2023 demonstrated multiple calcified atherosclerotic plaques along the length of both common carotid arteries with less than 50% bilateral stenosis. This residual macrovascular disease reflects atherosclerosis established during the decades of severe pre-transplant hypercholesterolaemia—a manifestation of the ‘cholesterol–year burden’ concept described by Schmidt and Hoeg^12^.

#### Other Complications

• **Benign right breast lesion (2018):** Excised; no evidence of malignancy.
• **Osteoarthritis:** Managed conservatively.
• **Gastrointestinal reflux:** Managed with famotidine and Gaviscon.

### 6. Immunosuppression trajectory

The patient’s immunosuppression history reflects the tension between maintaining adequate rejection prophylaxis for the cardiac allograft and minimising the nephrotoxic, metabolic, and skeletal burden of long-term immunosuppression. As noted by Ibrahim et al.^11^, the cyclosporin trough levels maintained throughout follow-up were substantially lower than those used in isolated cardiac transplantation, consistent with the attenuating effect of hepatic immunological privilege on allograft immune reactivity. The conversions to sirolimus in 2007 (renal-sparing intent), and subsequently back to ciclosporin in 2013–2014 (rejection control), reflect this clinical tension. At the 14 May 2026 clinic review, ciclosporin was adjusted from 75 mg twice daily to 75 mg AM/50 mg PM following an elevated trough of 155 ng/ml; target range 100–120 ng/ml.

**Table:** 

**Period**	**Immunosuppressive regimen**
1990–1994	Cyclosporin(a) at higher-than-standard doses; initial trough levels 550–664 ng/ml^11^
1994–2007	Cyclosporin(a) continued at progressively lower doses than isolated cardiac transplant recipients (years 5–10: 87–107 ng/ml; years 10–12: 53–80 ng/ml; years 12+: 35–50 ng/ml), reflecting hepatic immunological privilege. MMF 250 mg twice daily added from 2002^11^
2007–2013	Conversion from cyclosporin(a) to sirolimus 3 mg once daily (plus MMF 250 mg twice daily), in attempt to preserve renal function^11^
September 2013	Biopsy-confirmed Grade 1R cardiac rejection on sirolimus. Pulse methylprednisolone. Conversion to ciclosporin initiated
June 2014	Presumed second rejection episode (negative biopsy; response to three-day pulse methylprednisolone consistent with false-negative result). Ciclosporin consolidated
Current (2026)	Ciclosporin (Neoral) 75 mg AM / 50 mg PM (dose reduced from 75 mg BD at 14/05/2026 clinic following trough of 155 ng/ml; target 100–120 ng/ml); MMF 250 mg twice daily. Atorvastatin 10 mg od; Ezetimibe 10 mg od

### 7. Status at key intervals

**Table:** 

**Interval**	**Summary status**
3 weeks post-op (1990)	Mild cardiac rejection. Intensive prednisolone therapy. Full recovery.
1 year (1991)	LDL-C normalised. Cardiac and hepatic graft function satisfactory. Tendon xanthomata regressing.
5 years (1995)	Excellent ongoing graft function. Lipid control maintained. Cyclosporin dose progressively reduced.
10 years (2000)	Long-term stability. Renal function under surveillance as cyclosporin exposure accumulates.
15 years (2005)	Progressive renal impairment developing. Both grafts functional.
17 years (2007)	Conversion from cyclosporin to sirolimus-based regimen in attempt to preserve renal function. Creatinine 285 μmol/L^11^.
20 years (2010)	EF 68%; fractional shortening 30%; coronary calcification score 0. LDL-C 4.3 mmol/L on atorvastatin. End-stage renal failure established; haemodialysis commenced^11^.
23 years (2013)	Biopsy-confirmed Grade 1R cardiac rejection on sirolimus. Pulse methylprednisolone. Converted to ciclosporin. Full cardiac recovery.
24 years (2014)	Presumed rejection episode; pulse methylprednisolone (negative biopsy). Ciclosporin maintained.
28 years (2018)	Benign right breast lesion excised. No malignancy confirmed.
30 years (2020)	Both grafts functioning. Permanent haemodialysis via right brachiocephalic AV fistula.
33 years (2023)	Progressive CAV on surveillance imaging. Carotid Doppler: bilateral calcified plaques, <50% stenosis. AV fistula haemorrhage requiring surgical revision.
34 years (2024)	CTCA: progressive LAD calcification, increasing ostial circumflex stenosis, mild RCA and LM stenosis. EF 68%. Fall and femoral fracture: 3-month admission; pneumonia; deconditioning.
35 years (2025–2026)	MPS 05/2025: normal perfusion; EF 75%. Echo 05/2026: EF 64%. DSA negative. Ciclosporin dose adjusted. Bilateral arm weakness under investigation. Rotator cuff tear; frozen shoulder; left hip osteoporosis. Atypical chest symptoms under surveillance. Ongoing haemodialysis.

### Outcome measures

**Table:** 

**Outcome domain**	**Status at 35 Years**
Cardiac graft survival	In situ and functioning at 35 years. EF 64% (echo 05/2026); EF 75% on MPS (05/2025). Progressive CAV on CTCA (2024) without haemodynamic compromise.
Hepatic graft survival	Liver graft functioning throughout follow-up. No hepatic decompensation or graft failure documented.
Metabolic correction (LDL-C)	LDL-C reduced from 13 mmol/L (pre-operative) to 2.1 mmol/L (10 days post-operative); sustained long-term. Late residual hypercholesterolaemia on atorvastatin 10 mg od and ezetimibe 10 mg od, likely secondary.
Cardiovascular outcomes	Prior CVA. Progressive CAV (CTCA 2024). No inducible ischaemia (MPS 2022, 2025). No acute MI documented post-transplant. Residual carotid atherosclerosis (<50% stenosis bilaterally).
Rejection incidence	Two confirmed episodes across 35 years (3 weeks post-op; 2013). One probable episode (2014). Low overall burden; consistent with hepatic immunological privilege.
Renal function	End-stage renal failure secondary to CNI nephropathy. Permanent haemodialysis (Tue/Thu/Sat). Right brachiocephalic AV fistula.
Skeletal morbidity	Steroid-associated osteoporosis. Low-impact femoral fracture (2024); conservative management. Left hip osteoporosis pain limiting mobility. Rotator cuff tear and frozen shoulder.
Malignancy	Benign right breast lesion excised 2018. No confirmed malignancy across 35 years.
Functional status	Mobilising at home with walker; requires carers three times per week. Attends dialysis independently. Bilateral upper limb weakness limiting self-care. Physiotherapy ongoing.

## Discussion

This case, now observed over 35 years post-operatively, extends the translational narrative established by Ibrahim, El-Hamamsy, Barbir, and Yacoub at the 20-year mark^11^, updating each of the principal themes they identified—LDL metabolism, operational tolerance, and the limitations of chronic immunosuppression—and adding the important dimensions of skeletal morbidity and functional decline that emerge in long-term survivors.

### 1. Metabolic correction: Durability and mechanistic basis

The immediate normalisation of serum LDL-C from 13 mmol/L to 2.1 mmol/L following transplantation reflects the restoration of hepatic LDL receptor function by the donor allograft. This mechanism—documented first experimentally by Brown and Goldstein^17, 18^ and translated to clinical practice by Bilheimer, Goldstein, Grundy, Starzl, and Brown in the seminal 1984 case of a paediatric patient with HoFH who underwent liver transplantation^1^—forms the molecular foundation of CHLT as metabolic therapy for HoFH. As Ibrahim et al. noted, up to 80% of LDL receptors reside in the hepatic parenchyma; hepatic replacement therefore provides the dominant source of physiological LDL clearance^11^.

The persistence of LDL-C normalisation across more than three decades demonstrates that donor hepatic LDL-R function is durably maintained in the allograft, without the receptor dysfunction that characterises the native liver in HoFH. The late cholesterol elevation noted at the May 2025 clinic review warrants mechanistic consideration. Both atorvastatin (via HMG-CoA reductase inhibition and secondary LDL-R upregulation) and ezetimibe (via intestinal cholesterol absorption inhibition and secondary LDL-R upregulation) function through LDL-R-dependent pathways. Critically—and in contrast to the pre-transplant era when near-absent native receptor activity rendered these agents ineffective—the donor hepatic allograft now provides functional receptors through which both drugs can act. Residual hypercholesterolaemia in this context most likely reflects secondary dyslipidaemia driven by ciclosporin (which inhibits bile acid synthesis and reduces hepatic LDL-R expression), concurrent uraemia from end-stage renal failure, or modest late allograft LDL-R attrition, rather than recurrence of the primary genetic defect. Consideration of PCSK9 inhibitor therapy—which acts via LDL-R-dependent and partially independent mechanisms—may be appropriate given residual hyperlipidaemia on current dual therapy.

### 2. Cardiovascular outcomes: Residual risk and the cholesterol-year burden

Despite successful metabolic correction, this patient exhibits ongoing cardiovascular morbidity in the form of progressive CAV and a history of cerebrovascular accident, with residual bilateral carotid atherosclerosis documented on Doppler ultrasonography. This illustrates a fundamental translational limitation of CHLT: transplantation corrects the ongoing metabolic substrate for new atherosclerosis formation but cannot reverse pre-existing plaque burden accumulated during the decades of severe hypercholesterolaemia prior to surgery. The concept of ‘cholesterol-year’ burden—articulated by Schmidt and Hoeg^12^—captures this irreversibility: the degree of calcific atherosclerosis correlates with cumulative lifetime cholesterol exposure, and plaques established prior to transplantation persist in native vessels regardless of post-transplant lipid normalisation.

CAV represents an additional and distinct mechanism of coronary injury: immune-mediated diffuse intimal proliferation driven by alloreactive T-cell and antibody responses to the donor coronary endothelium, independent of traditional lipid-mediated atherosclerosis. The progression of CAV documented on CTCA in 2024—despite the hepatic immunological shield of CHLT—underscores that CAV surveillance remains mandatory even in combined transplant recipients. The preservation of myocardial perfusion on sequential MPS and maintained EF at 64–68% are nonetheless reassuring, indicating continued adequate functional reserve.

### 3. Operational tolerance: Mechanisms and long-term evidence

The low rejection burden in this patient—two confirmed episodes in 35 years, with extended periods on immunosuppressive doses substantially below those considered standard for isolated cardiac transplantation—constitutes compelling long-term clinical evidence for hepatic immunological privilege. Ibrahim et al. reviewed this phenomenon extensively at the 20-year mark, drawing on the experimental observations of Calne in pigs^3^, Kamada in rats^4^, and Starzl’s studies of microchimerism as a mechanism of donor-antigen tolerance^19, 20^. The liver’s anatomy promotes bidirectional interactions between donor and recipient leucocytes; its unique populations of antigen-presenting cells, sinusoidal endothelial cells, and Kupffer cells facilitate tolerogenic immune conditioning^21, 22^.

Additional mechanisms include induction of suppressor T-cell populations, immunological diversion from the cardiac allograft by the simultaneously transplanted liver, and the concept of ‘immune paralysis’ induced by the high antigenic load of combined organ transplantation—supported by observations that heart–lung recipients show less rejection than heart-only recipients^23^. Martinez-Llordella and colleagues identified transcriptional biomarkers of operational tolerance in liver allograft recipients, including expansion of γ*δ*TCR+ T cells and differential NK-cell activation^24^. The rejection episodes observed at 23 and 24 years post-transplant—during sirolimus-based immunosuppression—highlight an important caveat: hepatic immunological privilege attenuates but does not eliminate rejection risk, and immunosuppressive reduction must be approached cautiously.

### 4. Renal failure: The dominant long-term non-immunological complication

The development of end-stage renal failure secondary to CNI nephropathy represents the most significant long-term morbidity in this case, and a critical translational lesson for the management of long-term CHLT survivors. Ibrahim et al. noted that 40% of patients surviving two decades post-transplantation have renal failure or have received a renal transplant, observing that careful management had, at the 20-year mark, negated the need for renal replacement therapy despite creatinine rising from 135 to 285 μmol/L^11^. They raised the question of whether conversion to a renal-sparing protocol should have occurred earlier, before irreversible renal damage had accumulated, noting that the reversibility of renal failure declines as a function of the length of the immunosuppressive regimen^11, 13^. This question has been answered in this patient’s subsequent course: despite the switch to sirolimus in 2007, renal failure progressed to end-stage, necessitating permanent haemodialysis.

This trajectory underscores the critical importance of early nephrology involvement, proactive monitoring of renal function from the first post-transplant decade, and—where clinically feasible—early CNI dose minimisation in CHLT recipients. The hepatic immunological privilege, by allowing lower-than-standard CNI doses, may partially attenuate but does not prevent CNI nephropathy in patients surviving beyond twenty years.

### 5. Skeletal morbidity: An under-recognised consequence of chronic immunosuppression

Skeletal morbidity represents a complication of long-term solid-organ transplantation that has received increasing attention but remains clinically underestimated. Chronic exposure to corticosteroids suppresses osteoblast activity, increases osteoclast-mediated bone resorption, impairs calcium absorption, and directly damages bone microarchitecture^14, 15^. Calcineurin inhibitors contribute independently by reducing osteoblast differentiation and promoting bone loss^15^. Renal failure compounds these mechanisms through secondary hyperparathyroidism, reduced renal vitamin D hydroxylation, and phosphate dysregulation^16^. Frailty—increasingly recognised as a consequence of cumulative immunosuppression, muscle wasting, and physical deconditioning—further amplifies fall risk and fracture vulnerability in ageing transplant recipients^25^.

In this patient, low-impact femoral fracture following a fall in late 2024—despite long-term bisphosphonate therapy—confirms established osteoporosis and marks a significant turning point in functional trajectory. Conservative fracture management due to infection risk necessitated a three-month hospital admission complicated by pneumonia and deconditioning. The patient now requires a walker, carers three times per week, and is unable to feed herself independently due to upper limb weakness. These events collectively illustrate how skeletal and functional complications, emerging in the fourth decade post-transplant, may become the dominant determinants of quality of life even when cardiac and hepatic grafts remain functional. This case reinforces the importance of proactive bone health surveillance, early pharmacological bone protection, fall prevention strategies, and early frailty assessment in ageing transplant recipients.

### 6. Evolving pharmacological landscape: What is now available

The therapeutic landscape for HoFH has transformed substantially in the decades since this patient’s transplantation. At the time of surgery in 1990, the pharmacological armamentarium was limited to agents whose efficacy depended entirely on residual LDL-R activity. The subsequent development of PCSK9 inhibitors (evolocumab, alirocumab)—which prevent PCSK9-mediated LDL-R degradation and increase receptor recycling—provides meaningful LDL-C reduction in patients with partial LDL-R function, though efficacy is diminished in those with complete receptor loss^26^. Lomitapide, a microsomal triglyceride transfer protein inhibitor, and evinacumab, an anti-ANGPTL3 monoclonal antibody, act via LDL-R-independent mechanisms and can achieve substantial LDL-C reduction in true HoFH regardless of receptor status^26^. Inclisiran, a small interfering RNA targeting hepatic PCSK9 synthesis, offers twice-yearly dosing.

For the patient described in this report, these agents are now partially relevant: the donor hepatic allograft provides functional LDL-Rs, meaning LDL-R-potentiating agents such as PCSK9 inhibitors and statins now have a viable mechanistic substrate. Whether escalation to PCSK9 inhibitor therapy would be appropriate given residual hypercholesterolaemia on current dual therapy, and whether the renal failure status affects pharmacokinetics, warrants specialist review. This contrast between the therapeutic vacuum of 1990 and the current availability of injectable and RNA-based agents illustrates the dramatic translational progress achieved in this field across the lifespan of this patient’s post-transplant follow-up.

### 7. Translational lessons at 35 years

• **Metabolic correction:** CHLT provides near-definitive correction of HoFH through restoration of hepatic LDL-R function, with LDL-C normalisation sustained across 35 years. The modern pharmacological armamentarium—including PCSK9 inhibitors, lomitapide, and evinacumab—may further optimise residual hyperlipidaemia in CHLT recipients with functional donor hepatic allograft.
• **Irreversibility of pre-existing atherosclerosis:** CHLT corrects the ongoing substrate for new atherosclerosis but cannot reverse cholesterol-year burden accumulated prior to transplantation; cardiovascular surveillance must account for both residual native vascular disease and de novo CAV.
• **Hepatic immunological privilege:** Attenuates but does not eliminate cardiac rejection risk; long-term surveillance including endomyocardial biopsy capability and non-invasive imaging remains mandatory. Immunosuppressive regimen changes—even to renal-sparing agents—carry rejection risk for the cardiac allograft.
• **CNI nephropathy:** The dominant long-term non-immunological complication in patients surviving beyond twenty years. Early CNI minimisation strategies and nephrology co-management from the first decade are critical. Ibrahim et al.’s question—‘should the regimen have been switched earlier?’—remains unanswered by this case and warrants prospective study.
• **Skeletal morbidity:** An emerging dominant determinant of quality of life in long-term CHLT survivors. Proactive bone health surveillance, early pharmacological bone protection, fall prevention strategies, and early frailty assessment are essential in ageing transplant recipients.
• **Multidisciplinary care:** Long-term CHLT survival is associated with accumulating multi-system comorbidity spanning transplant cardiology, hepatology, nephrology, neurology, metabolic bone disease, pain management, and rehabilitation. Integrated, longitudinal multidisciplinary follow-up is essential.
• **Longevity is achievable:** This case confirms that CHLT can confer patient survival beyond 35 years, validating the strategy as a legitimate, potentially curative intervention for carefully selected patients with severe HoFH and advanced cardiovascular disease.

## Limitations

• This is a single-patient case report; findings cannot be generalised to the wider HoFH CHLT population.
• Complete serial lipid profile data at all predefined follow-up intervals are not fully available; selected time points are reported.
• Formal quality-of-life assessments using validated patient-reported outcome measures were not systematically captured.
• Confounding from dietary changes, lifestyle factors, and evolving co-prescriptions over three decades cannot be fully excluded.
• Some clinic correspondence contained an inconsistency in documented transplant year (1990 versus 1994 referenced in one letter), reconciled with the primary operative date of 13 September 1990 confirmed in the original case report^11^.

## Conclusion

This report documents one of the longest published follow-up periods for combined heart–liver transplantation performed for homozygous familial hypercholesterolaemia, with both cardiac and hepatic grafts functioning at 35 years post-operatively. The case demonstrates the extraordinary durability of metabolic correction achievable through hepatic replacement, confirming the translational promise first articulated at 20 years by Ibrahim, El-Hamamsy, Barbir, and Yacoub^11^ and supported by the foundational molecular discoveries of Brown, Goldstein, Bilheimer, and Starzl^1, 2, 17, 18^.

Cardiac graft function is preserved, the rejection burden across 35 years has been remarkably low, and hepatic graft function has remained intact—each consistent with the immunological advantage conferred by simultaneous hepatic transplantation. The principal translational lessons beyond the metabolic and immunological domains are the critical importance of anticipating and managing late complications: end-stage renal failure from CNI nephropathy, progressive cardiac allograft vasculopathy, steroid-associated osteoporosis with fragility fracture, and the accumulating burden of multi-system comorbidity in long-term survivors. The atypical chest symptoms, bilateral upper limb neuropathy, and skeletal morbidity identified at the most recent clinic review of May 2026 exemplify the complex, evolving clinical challenges that accompany survival into the fourth post-transplant decade.

As the therapeutic landscape for HoFH continues to expand—with injectable PCSK9 inhibitors, ANGPTL3 antagonists, and RNA-based therapies now available—the contrast with the therapeutic vacuum of 1990 that necessitated transplantation is stark. For this patient, the CHLT performed 35 years ago remains the defining intervention of her clinical trajectory, and its ongoing evaluation contributes rare and valuable outcome data to an under-documented field.

**Informed consent:** Written informed consent for long-term follow-up and publication was obtained from the patient. All identifying information has been removed to ensure anonymity.
